# Brain activity in response to the touch of a hand on the center of the back

**DOI:** 10.1371/journal.pone.0206451

**Published:** 2018-10-29

**Authors:** Ichizo Morita, Shigemitsu Sakuma, Junko Shimomura, Noriko Hayashi, Sueko Toda

**Affiliations:** 1 Japanese Red Cross Toyota College of Nursing, Toyota, Aichi, Japan; 2 Department of Fixed Prosthodontics, School of Dentistry, Aichi Gakuin University, Nagoya, Aichi, Japan; 3 Department of Health Science, Faculty of Psychological and Physical Science, Aichi Gakuin University, Nisshin, Aichi, Japan; 4 Tokai Gakuen University, Nagoya, Aichi, Japan; 5 Nagoya University of Arts and Sciences, Nisshin, Aichi, Japan; Tokai University, JAPAN

## Abstract

The aim of this study was to validate the possibility of using functional Near-Infrared Spectroscopy (fNIRS) to measure changes in cerebral blood flow in response to a hand being placed on a participant’s back, and to identify the areas of enhanced activity in the brain. Nineteen female adult volunteers participated in the study. An experienced school nurse touched the center of the participant’s back between the shoulder blades with the palm of her hand. Cerebral blood volume dynamics were measured with a 52-channel fNIRS system. Significantly higher oxygenated hemoglobin (oxy-Hb) concentration levels were recorded by channels 11, 14, 21, 22, 24, 32, 35, 45, 46, and 49 during the touching period than during the resting period. These channels indicated enhanced activity in the supramarginal gyrus, the middle frontal gyrus, the superior temporal gyrus, and the inferior frontal gyrus. The ability to detect changes in cerebral blood flow using this method indicates the possibility of measuring changes in cerebral blood flow using fNIRS when a person is touched on the back. fNIRS has been shown to be useful for studying the effects of touch.

## Introduction

Health professionals in hospitals often use touch while caring for patients and clients. Many studies on the effect of mothers’ touching behavior have also been reported in the field of childcare and development [1–3]. Touch has been shown to improve the subjective assessment of physical and psychological functions in adult patients [4–7]. Generally, school teachers in Japan are not allowed to touch students. However, school nurses in Japan, called *yogo* teachers, can use their hands to touch students when they visit the school health room, to support them and help to solve their physical and psychological problems. The school nurse’s touch is expected to have the same effect on students as a mother’s touch has on an infant and a health professional’s touch has on patients and clients. However, there is insufficient evidence of the effectiveness of school nurses’ touch. Enrichment of such evidence is needed to provide school nurses with guidelines for touching students.

Functional near-infrared spectroscopy (fNIRS) is a non-invasive neuroimaging technique [8, 9]. fNIRS measures the concentration changes in oxygenated hemoglobin (oxy–Hb) and deoxygenated hemoglobin (doxy–Hb) in the cerebral cortex associated with neural activity [10]. Although fNIRS offers low spatial resolution compared with functional magnetic resonance imaging (fMRI) and positron emission tomography (PET), it has a high temporal resolution of 0.1 s [11]. Further, fNIRS is relatively tolerant of body movements and there are no restrictions on the subject’s position and location. For example, subjects do not need to lie in an enclosed space as with fMRI or PET, and are examined under more natural conditions throughout the experiment, allowing more freedom in task design [12]. Nevertheless, few studies have evaluated the possibility of using fNIRS to measure changes in cerebral blood flow in response to touch. Kida et al. reported that activation of the prefrontal cortex was measurable by fNIRS when wood, velvet, and a paintbrush were used to gently sweep the participant’s palm or forearm [13]. Bennet et al. showed that fNIRS detected brain responses when the right forearm and palm were touched by a watercolor brush [14]. In general, a school nurse will lightly touch a student’s back and shoulders through the clothes. No study has reported whether such stimulation causes an increase in brain activity measured by fNIRS.

Sensory information is input from the sensory area to the parietal association area and the premotor area, and higher-order processing is performed in the prefrontal area. In particular, a slow moving and gentle touch affects emotions by activating C-tactile afferents (CTs) [15, 16]. However, there are no reports on brain activity in response to a touch on the back while the recipient is clothed.

The first aim of this study was to validate the possibility of using fNIRS to measure changes in cerebral blood flow when a school nurse places a hand on a student’s back. The second purpose was to clarify which brain areas show enhanced activity in response to this type of touch.

## Methods

### Participants

Nineteen healthy female volunteers aged 20.8 ± 0.4 years (mean ± SD) participated in this study. All of the participants were university students. They were acquainted with the experienced school nurse (NH, female) who performed the intervention in this study, as she was one of their lecturers.

**Fig 1:**
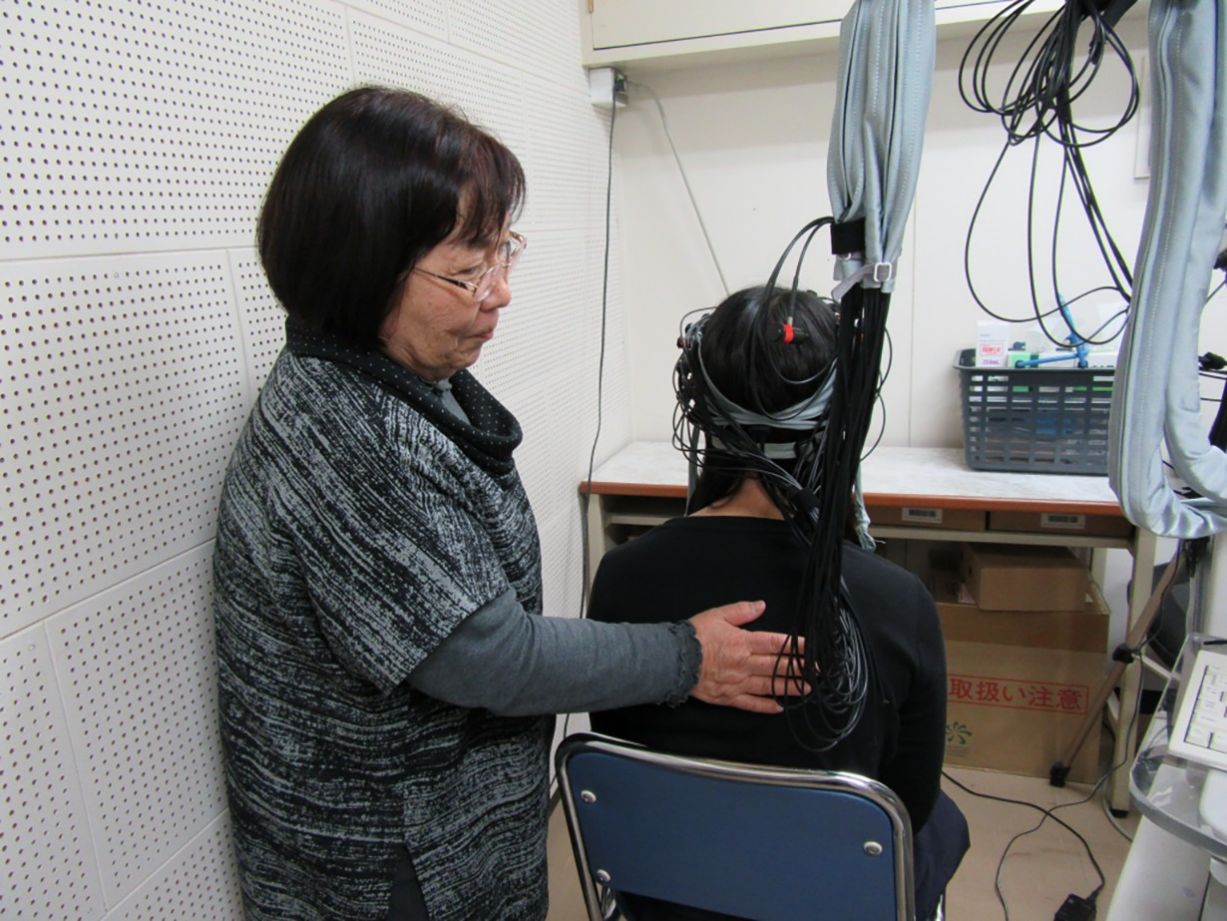
Position of the hand on the participant’s back.

This study was carried out with the approval of the Ethics Committee of Aichi Gakuin University, Faculty of Psychological and Physical Science, the Department of Health Science and the Department of Health and Nutrition (authorization number 1510). All participants gave their consent after receiving an explanation of the major purpose of the study. Written informed consent was obtained from all participants. The individual portrayed in Fig 1 has given written informed consent, as outlined in the PLOS consent form, to publish these case details.

### Experimental design

Participants sat on a chair and the experienced school nurse touched them on the center of the back following a block design procedure (Fig 1). One set of tactile stimuli was composed of two blocks of 30 seconds of rest (no touch) followed by 30 seconds of touch. Each participant received five sets of tactile stimuli, with a 30-second recovery time between sets. A preparation period of 90 seconds was set prior to a data collection. The procedure lasted 9 minutes in total.

Before the procedure began, participants were instructed to focus on a small black mark on the white wall about 2 meters away throughout the experiment. The school nurse stood on the left side of the participant and placed the palm of her right hand on the center of the participant’s back between the shoulder blades with a pressure of approximately 650 Pa. The hand remained as still as possible on the participant’s back for 30 seconds. This method of touch was named a “placing touch”.

### fNIRS data acquisition

**Fig 2:**
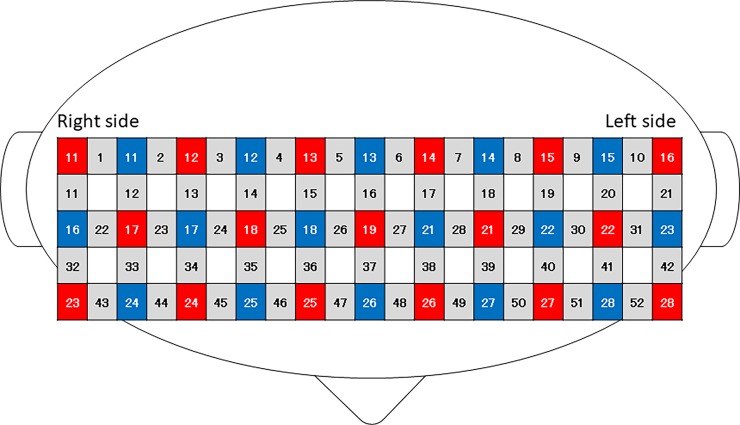
Schematic representation of the probe settings and measurement regions for 52-channel near-infrared spectroscopy (NIRS). Gray squares and numbers represent the measurement regions and channel numbers, respectively. Red and blue squares indicate near-infrared light emitter and detector positions, respectively.

Cerebral blood volume dynamics were measured with a 52-channel fNIRS system (ETG-4000; Hitachi Medical Co., Tokyo, Japan) equipped with 33 probes (17 emission and 16 detector probes). This machine has near-infrared laser diodes with two wavelengths (695, 830 nm), and calculates the concentration changes in oxygenated hemoglobin (oxy–Hb) and deoxygenated hemoglobin (deoxy–Hb) based on the modified Beer-Lambert law [17]. The near-infrared light is transmitted by an optical fiber to the emission probe positioned on the surface of the scalp, and the reflection of the light is received by the detector probe placed 3.0 cm away from the emission probe (Fig 2). The midpoint between an emission and a detector probe is regarded as the measurement position (channel). The sampling rate at each channel was 10 Hz. The probes (3×11) were mounted on the participant’s frontal and bilateral temporal regions. The lowest probe was located at the nearby T3–Fpz–T4 position according to the international 10–20 system for electroencephalogram recording.

### Statistical analyses

The oxy-Hb and deoxy-Hb data were used for the analyses, which were conducted for the 52 channels for each participant separately. Preprocessing of the fNRIS signals was performed by applying a 0.2 Hz low-pass filter. The averages for the 10 seconds at the end of rest and the first 10 seconds of the touch periods were calculated. Significant differences between the rest and touch periods were assessed using a paired t-test. The level of significance was set at p < 0.05 for each channel, with the after false discovery rate controlled by the Benjamini–Hochberg procedure (FDR_HB_) [18, 19], and those without a control. Statistical Package for the Social Sciences Version 19 was used for the analyses (IBM; Armonk, New York, NY, USA).

## Results

**Fig 3:**
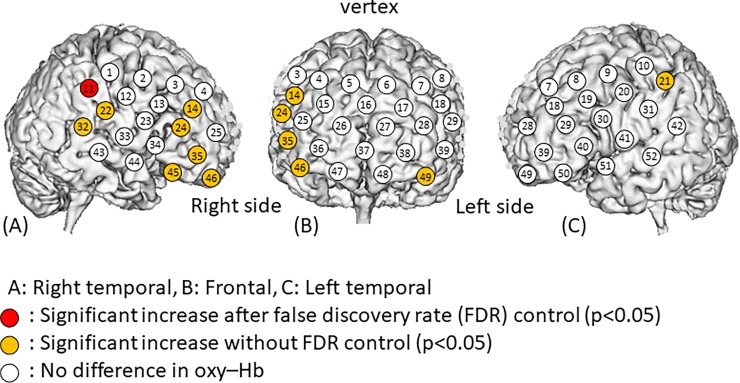
Changes in oxygenated hemoglobin (oxy-Hb) levels in response to touch. Oxy-Hb levels at each channel before and after touching were compared using a paired t-test. We found a significant increase in oxy-Hb at channels 11, 14, 21, 22, 24, 32, 35, 45, 46, and 49. After employing false discovery rate (FDR) correction via the Benjamini–Hochberg method, the increase in oxy-Hb remained significant at channel 11. The numbers in the figures represent channel numbers.

**Fig 4:**
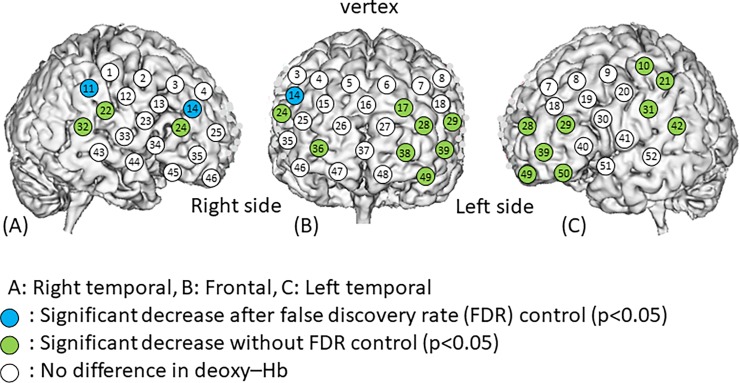
Changes in deoxy-Hb levels in response to touch. Deoxy-Hb levels in each channel before and after touch stimuli were compared using a paired t-test. We found a significant decrease in deoxy-Hb at channels 10, 11, 14, 17, 21, 22, 24, 28, 29, 31, 32, 36, 38, 39, 42, 49, and 50. After using FDR_HR_ correction, these decreases remained significant at channels 11 and 14. The numbers in the figures represent channel numbers.

Channel 11 showed the highest increase (0.027±0.022 m(mol/l)∙mm) in oxy-Hb concentration level in response to touch (Table 1) and channel 21 showed the second-highest increase (0.021±0.036 m(mol/l)∙mm). Channel 21 showed the largest decrease (-0.016±0.030 m(mol/l)∙mm) in deoxy-Hb concentration, followed by channel 11 (-0.012±0.012 m(mol/l)∙mm). The oxy-Hb concentration was significantly higher during the touch period than the rest period at channel 11 (p<0.05) with the FDR_HB_ control (Fig 3), while the deoxy-Hb concentration was significantly lower at channels 11 and 14 (p<0.05) after the FDR_HB_ control (Fig 4). The oxy-Hb concentration was significantly higher during the touch period than the rest period at channels 11, 14, 21, 22, 24, 32, 35, 45, 46, and 49 (p<0.05) without the FDR_HB_ control, while the deoxy-Hb concentration was significantly lower at channels 10, 11, 14, 17, 21, 22, 24, 28, 29, 31, 32, 36, 38, 39, 42, 49, and 50 (p<0.05) without the FDR_HB_ control.

**Table 1:** Concentration changes in oxy and deoxy–Hb levels in response to touch.

	Oxy–Hb		Deoxy–Hb	
	Mean	SD	Mean	SD
Ch1	0.010	0.032	-0.004	0.015
Ch2	0.001	0.020	-0.002	0.012
Ch3	0.004	0.019	-0.004	0.009
Ch4	0.006	0.026	-0.002	0.011
Ch5	0.002	0.024	-0.001	0.013
Ch6	0.002	0.021	0.000	0.012
Ch7	0.005	0.024	-0.001	0.010
Ch8	0.009	0.022	-0.005	0.011
Ch9	0.005	0.020	-0.001	0.007
Ch10	0.011	0.026	-0.008	0.011
Ch11	0.027	0.022	-0.012	0.012
Ch12	0.004	0.018	-0.001	0.011
Ch13	0.001	0.021	-0.005	0.013
Ch14	0.013	0.021	-0.008	0.009
Ch15	0.008	0.026	-0.003	0.011
Ch16	-0.005	0.024	-0.001	0.015
Ch17	0.004	0.023	-0.007	0.012
Ch18	0.006	0.023	-0.002	0.007
Ch19	0.006	0.025	-0.005	0.012
Ch20	-0.001	0.020	-0.002	0.011
Ch21	0.021	0.036	-0.016	0.030
Ch22	0.012	0.023	-0.007	0.013
Ch23	0.002	0.028	-0.003	0.018
Ch24	0.012	0.020	-0.005	0.008
Ch25	0.012	0.026	-0.005	0.010
Ch26	0.002	0.029	-0.004	0.011
Ch27	0.000	0.023	-0.003	0.011
Ch28	0.008	0.020	-0.006	0.010
Ch29	0.007	0.026	-0.004	0.009
Ch30	-0.002	0.042	-0.005	0.015
Ch31	0.002	0.035	-0.008	0.015
Ch32	0.017	0.027	-0.008	0.013
Ch33	0.015	0.038	-0.001	0.014
Ch34	0.002	0.028	-0.001	0.016
Ch35	0.012	0.023	-0.004	0.012
Ch36	0.012	0.028	-0.007	0.010
Ch37	0.001	0.036	-0.001	0.017
Ch38	0.008	0.026	-0.007	0.014
Ch39	0.006	0.028	-0.007	0.010
Ch40	-0.002	0.035	-0.007	0.018
Ch41	-0.007	0.034	-0.003	0.016
Ch42	0.003	0.026	-0.007	0.013
Ch43	0.019	0.045	-0.001	0.018
Ch44	0.009	0.032	-0.002	0.024
Ch45	0.018	0.029	-0.001	0.015
Ch46	0.017	0.032	-0.004	0.015
Ch47	0.016	0.038	0.000	0.015
Ch48	0.005	0.030	-0.001	0.008
Ch49	0.013	0.025	-0.009	0.012
Ch50	0.009	0.032	-0.007	0.011
Ch51	-0.008	0.032	-0.001	0.012
Ch52	-0.0002	0.030	-0.003	0.015

(Unit: m(mol/l)∙mm)

Changes in oxy (deoxy) Hb concentrations in each channel = first 10 seconds of the touch period data (the mean value of 19 participants)– 10 seconds at the end of the rest period data (the mean value of 19 participants).

## Discussion

We verified the ability of fNIRS to detect brain activation following a stimulus involving touching the back with the hand. The maximum change in oxy-Hb concentration from rest was previously reported to be 0.194 for pain stimulation to the gums [20] and 0.205 for volitional swallowing [21]. In the present study, the maximum change in oxy-Hb concentration with touch stimulation was 0.027, approximately 1/7 of that of a pain stimulus or at swallowing. Although such small changes in oxy-Hb concentration made statistical analysis difficult, we identified channels with significant changes.

Channels 11, 22, and 32 showed increased oxy-Hb levels, and were located on the primary somatosensory cortex, corresponding to the supramarginal gyrus and the postcentral gyrus. A study examining the proportions of the human brain dedicated to processing sensory functions reported that the area of the primary somatosensory cortex related to the body trunk was smaller than that for the hand or face [22]. Further, a stimulation involving placing touch only causes a small change in cerebral blood flow. Thus, further studies are required using a stronger stimulation to the back, such as with a stroking touch and a patting touch, which may cause greater changes in cerebral blood flow.

In the present study, brain blood flow activation following placing touch stimulation caused greater changes in the right versus the hemisphere of the brain (Fig 3). As described, the activated areas included the primary somatosensory cortex (supramarginal and postcentral gyri), as well as the middle frontal gyrus (channels 14, 24, 35, and 46) and the inferior frontal gyrus (channel 45). Paired t-tests revealed a trend at these channels for oxy-Hb to increase. Thus, the right hemisphere should be used for region of interest measurement of brain activity by touch stimulation to the middle frontal gyrus (channels 3, 4, 14, 15, 24, 25, 35, 36, and 46), inferior frontal gyrus (channel 45), postcentral gyrus (channel 12), precentral gyrus (channels 2, 13, and 23), supramarginal gyrus (channels 1 and 11), and superior temporal gyrus (channels 22, 32, and 33).

In the present study, only channel 11 showed a significant increase in oxy-Hb after the FDR_HB_ control as well as a significant decrease in deoxy-Hb. A similar pattern of changes in NIRS parameters is commonly observed in normal adult subjects [23]. Channel 11 measured activity in the supramarginal gyrus, which is part of the somatosensory association cortex and contains part of Wernicke’s area, which is involved in understanding language. Recently, the right supramarginal gyrus was also reported to be crucial for overcoming emotional egocentricity [24].

Channel 45 showed a significant enhancement in oxy-Hb, with a slight decrease in deoxy-Hb. Similar changes were observed in channels 11 and 21, and it was the most common pattern of change overall. The orbitofrontal cortex, in the lower area of the orbital part of the inferior frontal gyrus corresponding to channel 45, is considered to be involved in the integration of sensory information, decision making, and action planning [25]. In addition, Fellows et al. suggested that the orbital prefrontal cortex is involved in the adjustment of behavioral flexibility and emotion [26].

Channel 14 also showed the most common pattern of change, an increase in oxy-Hb and a decrease in deoxy-Hb. This channel measured activity in the right dorsolateral prefrontal cortex, which is related to working memory and plays a monitoring role in solving problems [27, 28]. Stuss et al. reported that patients with damage to the right dorsolateral prefrontal cortex showed impaired performance on the Wisconsin Card Sorting Test [29], which is designed to evaluate concept formation and conversion ability [30]. Hyperactivation of this area contributes to the switching of consciousness and the formation of intention. Touching the back might encourage the resolution of emotions, and thus help to heal students who visit the school health room.

fNIRS signals include various artifacts such as the effect of heart rate, respiration rate, and Mayer waves [31]. As these artifacts may affect the analysis of fNIRS data, we applied a 0.2 Hz low-pass filter to suppress heart rate signals. We also performed 90s cycle measurements to produce a phase shift between the 10-s Mayer waves. Recent studies also suggest that extracerebral blood flow may affect fNIRS signals [32]. Funane et al. [33] examined the effect of layers of deep tissue (cerebral) and shallow tissue (scalp, skin) on fNIRS signals, and found that 60%–70% of the signals reflected deep tissue responses. In addition, simultaneous fNIRS, fMRI, and laser Doppler flowmeter measurements showed that NIRS signals were significantly correlated with blood-oxygen-level-dependent signals in the gray matter, rather than with signals in the soft tissue or laser Doppler flow signals. Thus, we consider the main source of the fNIRS signals in our study to be related to cerebral blood flow. However, further studies are needed to examine the effect of skin blood flow on measurement data.

fNIRS measurements can also be sensitive to artifacts unrelated to brain activity, such as poor optical fiber contact caused by bodily movement or changes in blood flow associated with muscle activity [34]. Therefore, it is unclear whether the artifacts are uncontaminated. We obtained data using a 52-channel probe, and no clear artifacts were observed in the oxy-Hb waveforms obtained using this measurement.

In a previous study, stroking with a soft brush on the right forearm activated the contralateral primary and bilateral secondary somatosensory cortices [15]. This phenomenon was explained as a reaction to CT stimulation. In our study, the bilateral channels near the somatosensory cortex (11 and 22) were activated in response to touch. In this study, the interviewer touched the center of the back to simulate the real-life action, and this may have caused bilateral activation. However, in future, examination of the difference between the touched position and left and right brain activity should clearly separate the touch on the left and right.

CT afferents respond vigorously to slow and light stroking and are found only in hairy skin [35]. Stroking at 3 cm/sec at a temperature of 32°C produced the highest CT responses and pleasantness ratings [36]. We did not collect information on the temperature of the touched area in the current study. However, in our previous study using a similar protocol, the temperature of the touched area ranged from 32°C to 34°C and the pleasantness rating at 32°C was higher than that at 18°C and 42°C.

In our study, the interviewer touched the participants through their clothes. Future studies should investigate whether the response to a direct touch on the skin differs from a touch through clothing.

Several limitations of this study should be noted, however. The study was conducted on adult participants aged approximately 20 years; however, visitors to the school health room are elementary to high school students. Children and adults may react differently to touch. Moreover, all of the participants were female. In Japan, almost all school nurses are female, and those who touched the participant’s back in this study were female. The reaction may differ depending on the gender of the person touching and being touched. It may also depend on the relationship between them; in this study, which simulated the touch by a school nurse, the participants knew the person who touched them.

In this study, we evaluated changes in blood flow in the brain in response to a placing touch. Although changes in blood flow in the brain may differ with different kinds of touch, such as patting and stroking, these were not examined in this study.

## Conclusions

The findings of this study demonstrate that it is possible to use fNIRS to measure changes in cerebral blood flow when a hand is placed on a person’s back. The results showed slightly enhanced activity in the supramarginal gyrus, the orbitofrontal cortex in the lower area of the orbital part of the inferior frontal gyrus, and the dorsolateral prefrontal cortex in response to being touched on the back.
